# Structural MRI substrate of long-duration response to levodopa in Parkinson’s disease: an exploratory study

**DOI:** 10.1007/s00415-021-10550-5

**Published:** 2021-04-17

**Authors:** Giulia Donzuso, Giorgia Sciacca, Cristina Rascunà, Calogero E. Cicero, Giovanni Mostile, Alessandra Nicoletti, Mario Zappia

**Affiliations:** Department of Medical, Surgical Sciences and Advanced Technologies “GF Ingrassia”, University of Catania, Via Santa Sofia 78, 95123 Catania, Italy

**Keywords:** Parkinson’s disease, L-dopa, Long-duration response (LDR), Magnetic resonance imaging (MRI), Voxel-based morphometry (VBM)

## Abstract

**Objective:**

The long-duration response (LDR) to L-dopa is a sustained benefit deriving from chronic administration of therapy to patients with Parkinson’s disease (PD). Almost all patients with early PD may develop the LDR to L-dopa, even if some patients could not at given dosages of the drug. Aim of this exploratory study is to investigate whether a neuroanatomical substrate may underlie the development of the of LDR using structural magnetic resonance imaging (MRI) and voxel-based morphometry (VBM) analysis.

**Methods:**

Twenty-four drug-naïve PD patients were enrolled and underwent a baseline 3D T1-weighted structural brain MRI. Then, a treatment with 250/25 mg of L-dopa/carbidopa every 24 h was started and, after 2 weeks, LDR was evaluated by movement time recordings.

**Results:**

After 2 weeks of continuative therapy, 15 patients (62.5%) showed a sustained LDR (LDR +), while nine patients (37.5%) did not develop a sustained LDR (LDR −). VBM analysis on MRI executed before treatment showed changes of gray matter in precentral and middle frontal gyri in patients subsequently developing a sustained LDR with respect to those patients who will not achieve LDR.

**Conclusions:**

Parkinsonian patients who will develop a LDR to L-dopa may present, before starting treatment, peculiar structural conditions in cortical areas involved in motor control. Our exploratory study suggests that some cortical structural changes may predispose individual patients for developing the LDR to L-dopa.

**Supplementary Information:**

The online version contains supplementary material available at 10.1007/s00415-021-10550-5.

## Introduction

L-dopa still remains the best available symptomatic treatment for Parkinson’s disease (PD) and therapy response is a pivot point of diagnostic criteria and differential diagnosis [1]. It is well-known that the therapeutic response to L-dopa consists of two components: the short-duration response (SDR), an improvement of the clinical condition lasting few hours following the administration of a single dose of L-dopa, and the long-duration response (LDR), a sustained antiparkinsonian benefit deriving from prolonged administration of L-dopa, lasting days after discontinuation of treatment [2]. Different mechanisms for developing LDR have been proposed, such as peripheral and central pharmacokinetics phenomena or processes involving transduction pathways or receptors sensitization [3], but findings support the hypothesis that the LDR is dependent on a storage compartment that slowly releases dopamine synthesized from the exogenously supplied L-dopa [4, 5].

In clinical practice, the LDR is not usually taken into account and, in the earlier phases of PD course, L-dopa is often arbitrarily scheduled several times a day, without considering its long-duration effect. Indeed, it has been demonstrated that a multiple daily intake of small doses of L-dopa did not provide a sustained LDR in early PD patients [5], while full doses of 250 mg, once or three times per day, led to a sustained LDR in 84% of early PD patients [4]. Nevertheless, the achievement of a satisfactory LDR was independent from clinical features at baseline or from the SDR to an acute L-dopa test, but it seemed related to the interval time of L-dopa doses administration [4]. Considering that PD patients achieving a sustained LDR could be indistinguishable by clinical measures at baseline, the different initial response to L-dopa therapy and the different maintenance of LDR overtime could be supported by the presence of predisposing structural or functional features involving brain networks.

In the present study, we aimed to explore the hypothesis that peculiar structural brain conditions could underlie the development of the LDR to L-dopa in PD. To accomplish this purpose, we planned an exploratory study in which drug-naïve PD patients underwent an MRI voxel-based morphometry analysis before starting a 15-day treatment period with L-dopa devoted for the achievement of the LDR to the drug.

## Methods

### Subjects and clinical assessment

Drug-naïve patients with a new diagnosis of idiopathic PD according to the diagnostic criteria of the UK Parkinson’s Disease Society Brain Bank [6] were enrolled at the Neurologic Clinic of the “University Hospital” in Catania. None of the patients had been previously exposed to any dopaminergic treatments or was under treatment with anticholinergic agents, antidepressants, or other centrally acting drugs. All patients gave their informed consent to the study approved from the local ethical committee.

### MRI data acquisition

At baseline, all PD patients underwent a brain MRI, according to our routine protocol with a 1.5 T unit (Signa HDxt, GE Medical Systems, Milwaukee, WI, USA). A 3D T1-weighted high-resolution spoiled gradient echo (SPGR) sequence with a 1.2-mm slice thickness and an isotropic in-plane resolution of 0.98 mm was acquired with the following parameters: repetition time 14.8 ms, echo time 6.4 ms, flip angle 25°, 115 slices, matrix size 256 × 256 and a field of view of 24 cm. In addition, all subjects underwent a T2-weighted and FLAIR images to exclude morphological abnormalities, vascular disease or intracranial lesions.

### Pharmacological responses

Patients underwent an acute challenge with 250/25 mg of L-dopa/carbidopa for assessment of the SDR serving for further evaluation of the LDR. Motor severity was assessed using the Unified Parkinson’s Disease Rating Scale-Motor Examination (UPDRS-ME). The motor response was also objectively assessed by movement time (MT), which was considered an objective and reliable indicator of bradykinesia and its L-dopa induced modifications [7].

Details of the acute levodopa test were described elsewhere [8]. Briefly, after an overnight fast, a single oral dose of 250/25 mg of levodopa/carbidopa was administered and a clinical and objective evaluation by MT was performed every 30 min for the first 2 hours and every 2 hours until the patient returned to the basal conditions. The magnitude of the SDR was calculated on MT values, for the most affected side (MAS), as the percentage between the difference of the patients’ basal values and peak values and the theoretical maximal improvement (the difference between the patients’ basal values and the lower range value for normal control subjects) by the formula (B–P) × 100/(B–N), where *B* is the basal value at unmedicated baseline, *P* is the peak value, and *N* is the lower range value for the normal controls. Then, a treatment with 250/25 mg of levodopa/carbidopa every 24 h was started and, after 2 weeks, the LDR to chronic treatment was evaluated on the MAS as the percentage of improvement with respect to the maximal improvement observed for the SDR on the acute L-dopa test [4]. The LDR was calculated on the morning of the 15th day of treatment, before the morning dose intake, on MT values with the following formula: (B–X) × 100/(B–P), where *B* is the basal value at unmedicated baseline, *X* is the value on the 15th day, and *P* is the peak value of the acute L-dopa test. The achievement of a sustained LDR was considered when a value of at least 50% of the SDR was present after 15 days of treatment. This procedure has been reported to be a standardized treatment for achieving a sustained LDR [4, 7]. According to the achievement of a sustained LDR, patients were categorized as LDR + (those who developed a sustained LDR) and LDR − (those without a sustained LDR).

### Voxel-based morphometry (VBM)

We performed a voxel-based analysis investigating gray matter (GM) volume changes. Data were processed using the SPM8 software (http://www.fil.ion.ucl.ac.uk/spm), where we applied VBM implemented in the VBM8 toolbox (http://dbm.neuro.uni-jena.de/vbm.html) and incorporated the DARTEL toolbox that was used to obtain a high-dimensional image registration and normalization. Images were bias-corrected, tissue classified obtaining GM, white matter (WM) and cerebrospinal fluid (CSF) volume and registered using linear (12-parameter affine) and non-linear transformations, within a unified model. Subsequently, the warped GM segments were affine transformed into MNI-152 space and were scaled by the Jacobian determinants of the deformations (modulation). Finally, the modulated volumes were smoothed with a Gaussian kernel of 8-mm full width at half maximum (FWHM) [9].

### Statistical analysis

Data were analyzed using SPSS (version 23.0; IBM Corp., Armonk, NY). Quantitative variables were described using means and standard deviations, qualitative variables were described as proportions. Differences between means were evaluated by the unpaired *t*-test. In case of not a normal distribution appropriate non-parametric tests were performed. Differences between proportions were evaluated by the Fisher’s exact test.

### Image analysis

Voxel-wise comparison of GM density in selected regions of interest (ROIs) was performed. A full factorial model analysis (two-way ANOVA test) for the two PD subgroups was performed. To examine the main effect of LDR on GM density as well as the main effect related to the hemisphere contralateral to the clinical MAS, both LDR and hemisphere contralateral to MAS were considered as factors in the statistical model. Based on previous studies [10–14] nine cortical and subcortical ROIs, involved in motor control and response to dopaminergic therapy, were selected bilaterally: basal ganglia (caudate, putamen, pallidum), supplementary motor area, precentral gyrus, postcentral gyrus, superior, middle and inferior frontal gyri. All ROIs were created with the “aal.02” atlas included in the Wake Forest University Pickatlas software version 1.04 (http://www.fmri.wfubmc.edu/download.htm). All analyses were thresholded using correction for multiple comparisons inside ROIs (*p* < 0.05 family wise error, FWE at peak level with number of voxels > 10, considering the exploratory nature of the study). Age and total intracranial volume (ICV) were included in the model as covariates. In addition, GM signal intensity values for each significant cluster and for single patient were extracted using MarsBar tool integrated in SPM8.

## Results

### Demographics and clinical characteristics

Twenty-four drug-naïve right-handed PD patients were enrolled (Table 1). They had a relatively short duration of disease and a mild to moderate PD, expressed as UPDRS-ME scores and Hoehn and Yahr stage. The majority of patients were more affected from the left side. All patients presented a clear SDR to the acute levodopa test.

**Table 1:** Demographics and clinical characteristics of 24 patients with Parkinson’s disease

	Patients *N* = 24	LDR + *N* = 15	LDR − *N* = 9	*p*-value
Sex, men (%)	16 (66.6%)	9 (60%)	7 (77.7%)	0.8
Age (years)	64.5 ± 6.9	62.7 ± 13.8	66.6 ± 4.2	0.3
Age at onset (years)	62.4 ± 7.5	63.2 ± 8.0	64.7 ± 4.8	0.3
Disease duration (years)	2.1 ± 1.1	2.2 ± 1.1	1.9 ± 1.3	0.5
MAS, right (%)	11 (45.8%)	7 (46.7%)	4 (44.4%)	0.9
UPDRS-ME score	25.7 ± 9.1	24.0 ± 10.0	28.6 ± 6.8	0.2
Hoehn and Yahr stage	1.4 ± 0.5	1.3 ± 0.5	1.4 ± 0.5	0.7
SDR^				
MT basal, (msec)	362 ± 81	370 ± 92	348 ± 62	0.3
MT peak, (msec)	309 ± 66	318 ± 73	294 ± 51	0.4
Magnitude (%)	19.6%	18.9%	20.8%	0.5
LDR^a^				
MT basal, at 15th day (msec)	319 ± 70	308 ± 75	336 ± 58	0.4
MT peak, at 15th day (msec)	313 ± 72	307 ± 84	322 ± 48	0.7
Magnitude (%)	82.9%	121.7%	18.3%	0.00001

Data are means ± standard deviations

*MAS* most affected side, *UPDRS-ME* unified Parkinson’s disease rating scale-motor examination, *SDR* short duration response, *LDR* long duration response, *MT* movement time, *LDR* + patients with LDR, *LDR* −  patients without LDR

^a^Values recorded for the most affected side

After 2 weeks of administration of levodopa/carbidopa 250/25 mg once a day, 15 patients (62.5%) showed a sustained LDR, while nine patients (37.5%) did not achieve a sustained LDR. There were no significant clinical differences between the two subgroups, except for the LDR improvement at the 15th day of treatment.

### VBM results

**Fig. 1:**
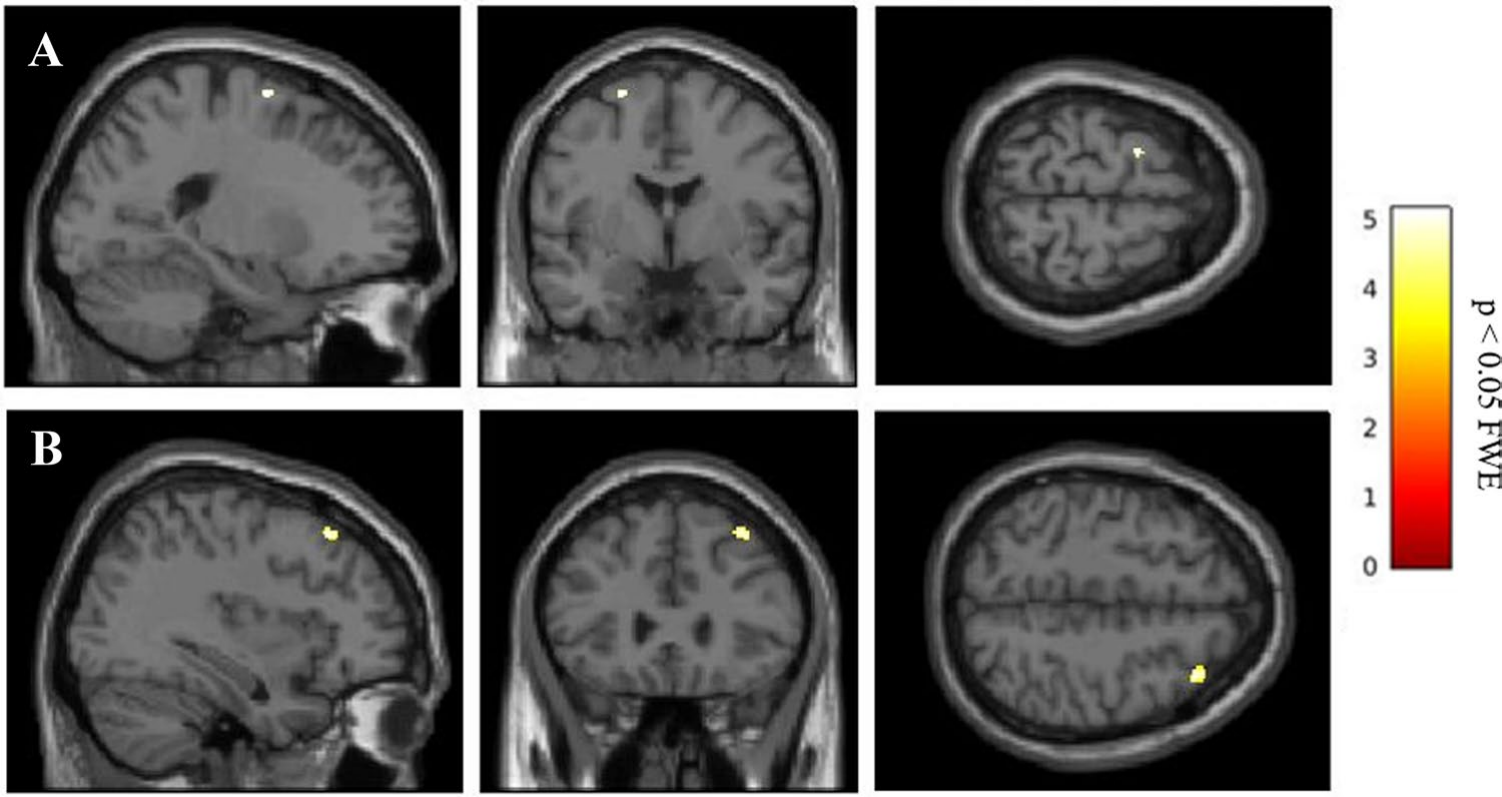
Clusters of GM volume changes in LDR + versus LDR − with right and left clinical MAS, involving **a** left precentral gyrus and **b** right middle frontal, respectively. *GM* gray matter, *MAS* most affected side, *LDR* + patients with LDR

**Fig. 2:**
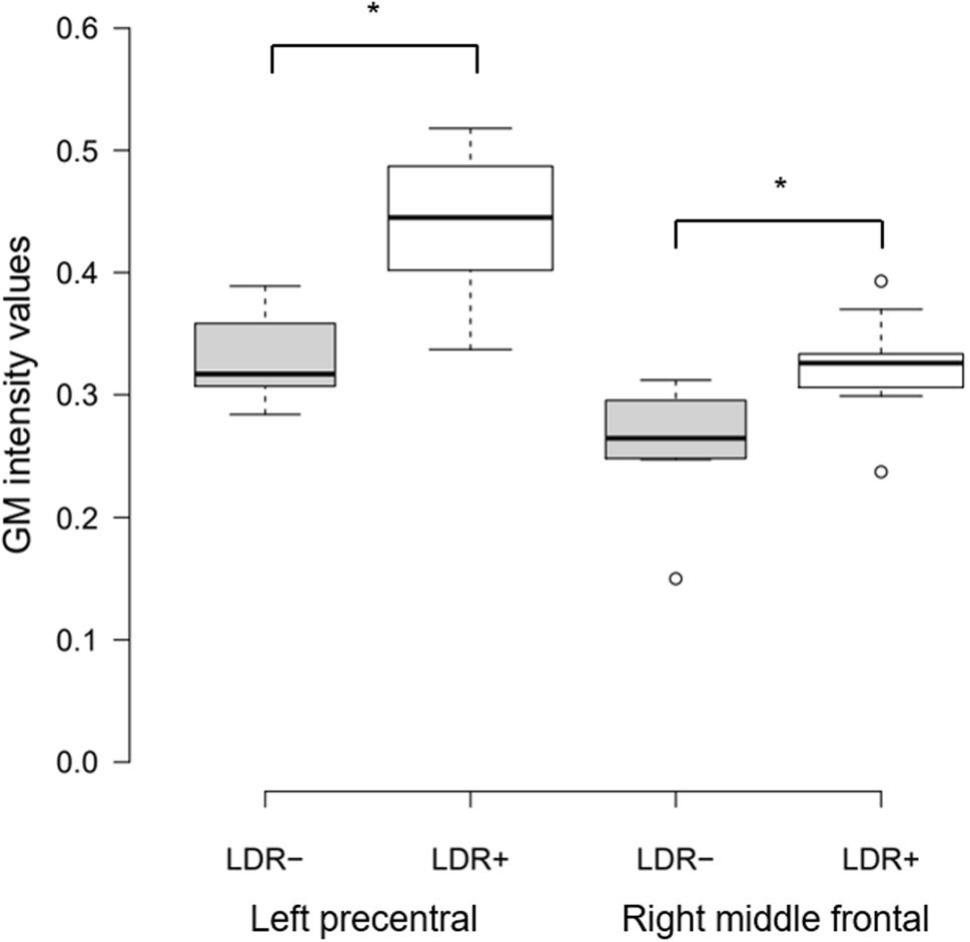
Boxplot showing differences in GM intensity values of the two significant clusters between LDR + and LDR − . Center lines show the medians; box limits indicate the 25th and 75th percentiles as determined by R software; outliers are represented by dots. * *t*-test between LDR + versus LDR − , *p* < 0.05. *GM* gray matter, *LDR* + patients with LDR, *LDR* − patients without LDR

The analysis of MRI obtained before beginning the treatment showed that no differences between LDR + and LDR − groups regarding whole brain volume of GM, WM and CSF (Supplementary Table 1). Nevertheless, analyzing the ROIs of selected basal ganglia and cortical areas, and considering the main effect of LDR, two significant GM clusters in left precentral gyrus (MNI local maxima: − 36, 3, 31; *F* = 27.20; *P*_FWE − ROI_ = 0.03) and in right middle frontal gyrus (MNI local maxima: 36, 27, 51; *F* = 22.56; *P*_FWE − ROI_ = 0.01) were evident (Table 2). When the analysis considered only the hemispheres contralateral to the clinical MAS, LDR + patients with a right clinical MAS (*n* = 7) showed a significant contralateral GM cluster in the left precentral gyrus (MNI local maxima: − 38, 2, 28; *t* = 5.13; P_FWE − ROI_ = 0.01), and vice versa, LDR + patients with left MAS (*n* = 8) had a significant GM cluster in right middle frontal gyrus (MNI local maxima: 36, 27, 51; *t* = 3.96; *P*_FWE − ROI_ = 0.04) (Fig. 1 and Table 2). No other significant differences were present outside the selected ROIs. Furthermore, GM intensity values of the two significant regions showing differences between groups (left precentral and right middle frontal gyri) were extracted. The individual data extracted for each patient confirmed the differences between PD-LDR + *versus* PD-LDR − (mean GM intensity values for left precentral gyrus: 0.437 ± 0.06 *vs* 0.330 ± 0.03, *p* = 0.0004; mean GM intensity values for right middle frontal gyrus: 0.323 ± 0.04 *vs* 0.260 ± 0.05, *p* = 0.008) (Fig. 2).

**Table 2:** Clusters with significant difference in patients with Parkinson’s disease grouped according to the achievement of the long-duration response to the L-dopa

	Hemisphere	Cluster size	Peak activation	Peak coordinates		
				X	Y	Z
Main effect group LDR + > LDR −						
Precentral gyrus	L	56	27.20*	−36	3	31
Middle frontal gyrus	R	44	22.56*	36	27	51
LDR + > LDR − with right clinical MAS						
Precentral gyrus	L	39	5.13^	−38	2	28
LDR + > LDR − with left clinical MAS						
Middle frontal gyrus	R	14	3.96^	36	27	51

The coordinates x, y and z refer to the anatomical location, indicating standard stereotactic space as defined by Montreal Neurological Institute

*L* left, *R* right, *LDR* long-duration response, *MAS* most affected side, *LDR* + patients with LDR, *LDR* −  patients without LDR

*p* < 0.05 family wise error (FWE), voxels > 10

*F-value

^*t*-value

## Discussion

Before starting treatment drug-naïve PD patients could present baseline GM differences in frontal cortical areas between patients who will develop a LDR to L-dopa and who will not after 2 weeks of continuative therapy. Specifically, we found GM changes involving precentral and middle frontal gyri in LDR + patients with respect to LDR − patients.

The LDR represents a considerable component of the clinical effect of L-dopa, but its underlying mechanisms remain poorly understood [3]. It has been estimated that the LDR represents a third to a half of the total motor function improvement during L-dopa therapy [15]. Recently, Cilia et al. [16] confirmed previous data and showed that the magnitude of the LDR was up to 65% of total motor improvement provided by L-dopa, independently of disease duration [16]. Moreover, the achievement of a sustained LDR did not appear to be related to some clinical characteristics or to the SDR obtained following an acute challenge with levodopa [4]. On the other hand, some patients could not develop a sustained response to dopaminergic treatments and the mechanisms underlying the failure to achieve the LDR are still unclear.

Our findings, even if preliminary considering the exploratory purpose of the study, suggest that some brain structural predisposition could underlie, or at least could be associated to, the development of the LDR to L-dopa. Recently, Ballarini et al. [17] demonstrated that a reduced GM density in the left temporoparietal operculum of PD patients was associated with a weaker response to dopaminergic therapy as compared to PD patients with a stronger response. Apart from that study, there is no evidence in the literature of data supporting the presence of structural brain changes leading to the development of a peculiar dopaminergic responsiveness. By contrast, many studies have investigated how the administration of L-dopa could induce functional brain changes of PD patients. Indeed, it is well-known that administration of L-dopa has a role in modifying network connectivity in PD patients at different stages of disease, as reported by several functional MRI studies showing improvement of the baseline connectivity in sensorimotor and striato-cortical networks [12, 18, 19]. Therefore, these observations may suggest a dynamic interplay between L-dopa administration and neural plasticity: indeed, L-dopa could modify network connectivity from one side, but structural changes could also predispose individual patients to a better L-dopa response such as a sustained LDR.

The involvement of frontal regions and their role in motor control and therapy response in de novo/early as well as moderate/advanced PD patients, has been broadly demonstrated [11, 13, 20–24]. Of note, the precentral gyrus is the anatomical location of the primary motor cortex, which is responsible for controlling voluntary motor movement on the body's contralateral side [25]. As regard to the middle frontal gyrus, it is well-known its main role for determining attentional processes; nevertheless, a recent meta-analysis on motor functional imaging in PD patients showed that the middle frontal gyrus could be also involved in motor control, indicating a functional remapping of the brain during motor execution [20]. This is in line with our findings, showing the involvement of the right middle frontal gyrus in PD patients with left clinical MAS and of the left precentral gyrus in those patients with right clinical MAS. Of course, the changes we observed in the frontal motor-related cortical areas and hypothesized to be the structural basis for future development of the LDR to L-dopa, could be simply related to the lateralization of brain structural changes associated to the contralateral clinical symptomatology. Nevertheless, the cortical organization has been reported to be not influenced by motor laterality in early PD [26], even if a cortical thinning in the motor areas of the hemisphere contralateral to the MAS has been also reported [27]. Thus, further studies are needed to understand the contribute of different peculiar frontal areas in relationship to the side of body involvement. Furthermore, it is unclear why our patients with right side involvement had a greater GM changes on the left precentral gyrus; whereas, patients with left side involvement showed a greater GM change in the right middle frontal gyrus. It is possible that handedness of PD patients [28, 29] and body side involvement could influence the brain structural organization of different cortical motor areas, but we have no evidence to suggest this hypothesis, also considering that our patients were all right-handed.

Our findings showing GM changes in peculiar frontal motor cortical areas, however, just indicated structural differences between patients LDR + and patients LDR − , but we cannot attribute these differences to an increase or to a decrease of cortical GM. In other words, both conditions could be hypothesized, i.e., a GM increase in patients who will develop a sustained LDR after treatment—maybe related to some compensatory mechanisms—or a GM decrease in patients who will not develop a LDR as consequence of peculiar cortical atrophy process.

Despite the intriguing findings described above, a major limitation of the current study is due to the relatively small sample size implicit in its exploratory nature that could affect the statistical power of our analysis, but the reliable VBM pipeline, and the strength of statistical threshold (correction for multiple comparisons inside ROIs (*p* < 0.05 family wise error, FWE at peak level with number of voxels > 10) could minimize the risk. Nevertheless, it should be considered that our patients were all drug-naïve and underwent a standardized short-term treatment aimed to develop a LDR to L-dopa, previously reported to be an adequate and standardized period of treatment for inducing the LDR at fixed doses given at peculiar inter-dose intervals [4]. On the other hand, we could not know in advance how many patients could achieve or not a sustained LDR to L-dopa and thus we planned an exploratory study in a small sample of patients. Thus, we are aware of the caution required in generalizing our results and the need to be reproduced in larger samples, but these findings could trace a starting point for future research.

In conclusion, the presence of changes of GM volume in brain cortical areas involved in motor control, could represent a structural predisposition leading to a sustained response to dopaminergic therapy.

## Supplementary Information

Below is the link to the electronic supplementary material.

Supplementary file1 (DOCX 14 KB)
