# Supplementary material for: Structural MRI substrate of long-duration response to levodopa in Parkinson’s disease: an exploratory study
Source: J Neurol. 2021 Apr 17;268(11):4258–64. doi: 10.1007/s00415-021-10550-5 (PMC8505285; doi:10.1007/s00415-021-10550-5)
Supplement: Supplementary file 1 — Supplementary file1 (DOCX 14 KB) [file 415_2021_10550_MOESM1_ESM.docx]

**Supplementary table 1.** General MRI features of 24 patients with Parkinson’s disease.

|  | **Patients**  **N=24** | **LDR+**  **N=15** | **LDR-**  **N=9** | **p-value** |
| --- | --- | --- | --- | --- |
| **MRI features (ml)** |  |  |  |  |
| **GM** | 569.7 ± 44.4 | 574.8 ± 51.6 | 561.0 ± 29.6 | 0.5 |
| **WM** | 514.2 ± 54.3 | 523.8 ± 54.4 | 498.0 ± 53.0 | 0.3 |
| **CSF** | 274.6 ± 47.4 | 270.3 ± 51.9 | 281.6 ± 40.6 | 0.6 |
| **ICV** | 1358.4 ± 107.7 | 1369.0 ± 120.1 | 1340.7 ± 87.0 | 0.5 |

Data are means ± standard deviations.

GM, grey matter. WM, white matter. CSF, cerebrospinal fluid. ICV, intracranial volume. LDR, long-duration response.

LDR+, patients with LDR. LDR-, patients without LDR.
